# Spent Coffee Waste as a Potential Media Component for Xylanase Production and Potential Application in Juice Enrichment

**DOI:** 10.3390/foods8110585

**Published:** 2019-11-17

**Authors:** Rajeev Ravindran, Gwilym A. Williams, Amit K. Jaiswal

**Affiliations:** 1 School of Food Science and Environmental Health, College of Sciences and Health, Dublin Institute of Technology, Cathal Brugha Street, D01 HV58 Dublin, Ireland; rajeev.ravindran@mydit.ie; 2 School of Biological Sciences, College of Sciences and Health, Dublin Institute of Technology, Kevin Street, D08 NF82 Dublin, Ireland; gwilym.williams@TUDublin.ie

**Keywords:** spent coffee waste, solid state fermentation, xylanase, fruit juice clarification, *Aspergillus niger*

## Abstract

In this study, spent coffee waste (SCW) was used as the sole carbon source for xylanase production in solid state fermentation mode using *Aspergillus niger*. A Box–Behnken design was constructed using three parameters viz. temperature, initial moisture content, and log number of spores to determine the optimal fermentation condition. The best fermentation conditions for xylanase production were found to be incubation at 30 °C with an initial moisture content of 70% and using an inoculum of 6.5 × 10^6^ spores/g of dry SCW. Furthermore, the design of experiments revealed that maintaining a medium composition of 0.2 g of yeast extract, 0.04 g of K_2_HPO_4_, and 0.03 g of MgSO_4_ increased xylanase production. Under optimised solid-state fermentation conditions an enzyme activity of 6495.6 IU/g of dry SCW was recorded, which was approximately 1.39-fold higher than that of control (4649 IU/g of dry SCW). The efficacy of the purified xylanase as a juice enrichment agent for strawberry, blueberry, and raspberry pulp was tested.

## 1. Introduction

Lignocellulosic residues are plant-based materials that are generated in large volumes by industries such as agriculture and forestry. They are rich in carbohydrates and lignin with small amounts other value-added compounds such as proteins, lipids, and polyphenols. They have gained considerable interest as a cheap carbon source in secondary fermentation processes. The polysaccharide fraction of lignocellulose can be broadly classified into cellulose and hemicellulose depending on homogeneity. Hemicellulose is the second most abundant carbohydrate fraction in lignocellulosic materials and constitutes 30% of the plant cell wall [1]. Xylan is one of the main components of hemicellulose. Xylan is degraded enzymatically by several bacterial and fungal species by synthesising a specific group of enzymes called xylanases.

Xylanases are hemicellulolytic enzymes that specifically degrade β-1,4-xylans found in lignocellulosic materials. Xylanases are commercially important from with wide applications in juice-wine clarification, food processing, paper pulping, etc. [2]. They are also used in combination with cellulase in the digestion of silage to improve nutritional qualities. Xylanase is commercially produced on a large scale by the utilisation of xylan as the key carbon source in fermentation. Xylan can be extracted from plant sources by acid/alkali treatment followed by separation techniques (such as chromatography) and precipitation [3]. However, enzymatic processes are commonly used abundantly to produce xylan [4,5]. The application of expensive enzymes can add to the cost of production of xylan. Xylan can arguably be replaced by cheap agricultural, lignocellulosic residues, which are abundantly available while also deploying enhanced fermentation strategies to reduce the enzyme production cost.

Most enzymes are commercially produced on a large scale by following submerged fermentation approaches due to ease of process control. However, solid state fermentation (SSF) is an alternative fermentation strategy that has numerous advantages such as high volumetric productivity, less effluent generation, simplified control instrumentation and equipment, and most importantly low capital investment and operational cost [2,6]. Filamentous fungi are excellent producers of lignocellulytic enzymes such as cellulases and xylanases and are perfect strains for enzyme production via solid state fermentation mode. Using such an approach combined with lignocellulosic residues combines holds out the possibility low-cost operation coupled by high enzyme titre [7]. Some of the common lignocellulosic residues employed in SSF-based enzyme production studies include rice bran, wheat bran, corn cob, and sugar cane bagasse [2]. 

Coffee waste is one such lignocellulose residue, which is expelled in abundance by the growing global coffee industry. Some of the by-products derived from coffee are coffee husk, silver skin, pulp, and spent coffee. Spent coffee is high in hemicellulose content, in addition to being a large reservoir of cellulose, lipids, proteins, and polyphenols. This makes spent coffee an ideal substrate for enzyme production [8]. Despite the high nutritional value, not many studies are available that utilises spent coffee in SSF mode for the production of xylanases. Murthy, et al. [9] published an interesting study where coffee by-products were used in SSF mode to produce xylanases employing *Pencillium* sp. CFR 303 as the enzyme producer.

In this study, we have attempted to utilise spent coffee waste (SCW) as the sole carbon source for xylanase production. *Aspergillus niger* ATCC^®^6275 was employed as the enzyme producing fungus. The process parameters for SSF mode were optimised by the application of response surface methodology. The subsequent xylanase produced was purified by a three-step strategy following which it was characterised. The purified xylanase was finally tested as a potential juice enrichment agent for three fruit pulp varieties.

## 2. Materials and methods

### 2.1. Materials

The spent coffee waste (SCW) employed for this study was kindly provided by a local Starbucks outlet in Dublin city. The SCW was washed and dried at 80 °C for 48 h in a hot air oven following which it was stored in a cool and dry place for further experiments. All other chemicals were purchased from Sigma Aldrich (Wicklow, Ireland).

### 2.2. Lipid Extraction from SCW

The total lipids were extracted from SCW by following the method of Ahahgari and Sargolzael [10]. Briefly, 10 g of SCW was weighed and transferred to cellulose thimble which was then stoppered with cellulose wool. A Soxhlet apparatus was set up with 100 mL of petroleum ether in a round bottom flask containing anti-bumping granules. Extraction was conducted for 6 h in reflux cycles or until a constant weight of petroleum ether-oil mixture was attained. The petroleum ether was then evaporated off leaving behind the freshly extracted SCW lipids. The weight of the lipids recovered was recorded and the lipid-free SCW was stored for further experiments.

### 2.3. Microorganism and Xylanase Production

*Aspergillus niger* derived from ATCC^®^6275 was obtained from the microbiology repository at the School of Food Science and Environmental Health, TU Dublin-City Campus. The microorganism was grown on potato dextrose agar (PDA) slants at 30 °C for five days and subsequently stored at 4 °C. The microbe was screened for xylanase production capability by cultivation on Czapek Dox agar plates supplemented with 1% xylan and incubated for 72 h at 30 °C. The plates were stained with 1% congo red for 20 min following which they were de-stained using and 1.0 M NaCl. The plates were checked for zones of clearances [11]. For fermentation purposes, the inoculum was prepared according to the protocol described by [12]. Briefly, spores from a sporulated slants culture were re-suspended in 0.1% sterile Tween-80. This was used a working cell bank from which dilutions were prepared for the experiments. 

### 2.4. Substrate Preparation and Solid-state Fermentation

SCW was subjected to organosolv pretreatment as mentioned in a previous study conducted at our facility [13]. Erlenmeyer flasks (250 mL) were used for solid-state fermentation and the growth medium comprised the following f (in g/g of dry SCW) CoSO_4_·7H_2_O, 0.01; CuSO_4_·5H_2_O, 0.05; KH_2_PO_4_, 0.5; and 1.0; industrial yeast extract, 0.05. Each flask was supplemented with five grams pre-treated SCW. The contents of the flask were autoclaved at 121 °C for 30 min following which it was inoculated with 1 mL of inoculum (~1 × 10^6^ spores/mL). The fermentation was carried out for 120 h, following which the enzymes were extracted [14]. 

### 2.5. Enzyme Extraction and Activity Assay

On completion of each fermentation experiment, the contents of each flask were re-suspended by addition of citrate buffer (0.05 M, pH 5.0). A solid-to-liquid ratio of 1.0 g initial dry substrate/12 mL of buffer was maintained for xylanase extraction. This mixture was homogenised for 20 min and centrifuged for 1200 rpm for 30 min at 4 °C. The solids–liquid separation was conducted by centrifugation, followed by a filtration polishing step through a Whatman no. 1 filter paper to remove any residual particulate matter. The filtrate was then assayed for xylanase activity. Xylanase activity was estimated as follows: 50 μL of enzyme solution was mixed with 100 μL of 1.0% Birchwood xylan (≥90% purity) prepared in 0.05 M citrate buffer (pH 4.8). The mixture was incubated for 20 min at 50 °C [14]. This was followed by determination of reducing sugar formation employing the dinitrosalicylic acid method [15]. Xylose was used as a standard. One unit was defined as the amount of enzyme that releases 1.0 μmol of xylose in 1 min under assay conditions.

### 2.6. Optimisation of Process Parameters

#### 2.6.1. Optimisation of Physical Parameters

In this study the three physical parameters pertaining to solid state fermentation *viz.* temperature, moisture content, and inoculum size (log number of spores) was optimised by the application of response surface methodology. A Box–Behnken design was employed for this purpose involving the three factors and three different levels for each factor (Table 1). A total of 15 experiments were conducted in triplicate and the average xylanase activity determined. A second-order polynomial equation was generated and analysed by the statistical software Statgraphics Centurion XV software version 15.1.02 (StatPoint Technologies Inc. Warrenton, VA, USA). The general form of the polynomial equation is given below:

*Y_ί_ = β_o_ + Σ β_ί_ Χ_ί_ + Σ β_ίί_ Χ_ί_^*2*^ + Σ β_ίj_ Χ_ί_ Χ_j_,* (1)

where *Y_ί_* is the response variable while *β_o_* is the offset term and *β_ί_*, *β_ίί_*, and *β_ίj_* are the linear, quadratic, and interaction coefficients, respectively [16].

The model was subjected to statistical analysis by performing analysis of variance (ANOVA). This analysis also encompassed Fischer’s *F-*test (overall model significance), the associated probability, p(F), correlation coefficient R, and determination coefficient R^2^, which indicates the good fit of regression model.

#### 2.6.2. Plackett–Burman Design for Identification of Significant Variables

A Plackett–Burman design was adopted to investigate the effect of medium components on xylanase production using SCW. Plackett–Burman is essentially a two-factorial design, which identifies the physico-chemical parameters required to increase the levels of xylanase production. This design of experiments strategy assumes that there are no interactions between variables. The variables are investigated at two levels, −1 (low level) and +1 (high level) spaced by a large interval. The experimental design conducts *n* + 1 screening trials for n variables. In this study, 10 potential media components *viz.* KH_2_PO_4_, NaCl, MgSO_4_, yeast extract, peptone, (NH_4_)_2_SO_4_, NH_4_Cl, CaCl_2_, FeCl_3_, and KCl were included to determine which enhanced the production of xylanase by *A. niger* ATCC^®^6275 via solid state fermentation (Table 2). 

Minitab^®^17.0.1. was used to generate the experimental design. The effects of individual variables on the production of xylanase is calculated by the following equation:

*E= (ΣM_+_ − ΣM_−_)/N,* (2)

where *E* is the effect of the parameter and M_+_ and M_-_ are responses (xylanase activity) of each experimental trial where parameters are at their highest (M_+_) and lowest (M_−_) levels while *N* is the number of trials.

#### 2.6.3. Optimisation of Media Composition

As with optimisation of physical parameters for SSF optimisation, a Box–Behnken design was adopted to optimise the concentration of media components identified in the Plackett–Burman screening study. Three parameters and three levels were taken into account to generate the model. All statistical analyses were carried out using Statgraphics Centurion XV software version 15.1.02 (StatPoint Technologies Inc. Warrenton, VA, USA).

### 2.7. Purification of Xylanase

Xylanase was purified using a modification of the protocol devised by [17]. Crude xylanase obtained after solid state fermentation was subjected to 65% *w*/*v* (NH_4_)_2_SO_4_ saturation and incubated overnight at 4 °C. The solution was then centrifuged at 15,000 rpm for 30 min. The precipitates recovered were dissolved in 0.05 M citrate buffer (pH 6.0). Desalination was performed by dialysis using a cellulose tubing (molecular mass cut-off 10 kDa) against citrate buffer for 24 h. The buffer was changed frequently. The xylanase was further concentrated by diafiltration using a 10 kDa MWCO Amicon Ultra-15 centrifugal filter unit (Millipore, Darmstadt, Germany) and centrifugation at 5000 rpm for 10 min at 4 °C. The final purification step involved anion exchange chromatography using a DEAE-cellulose column (1.5 cm × 6.0 cm; flow rate 30 mL/h), which was equilibrated with 0.05 M citrate buffer (pH 6.0). After elution of unbound proteins, the retained fraction was collected with a step elution of varying concentrations of KCl (0.1~0.5 M) in citrate buffer and 3 mL fractions collected. The active fractions were pooled and concentrated by ultrafiltration (10 kDa cut-off).

### 2.8. Application of Xylanase for Fruit Juice Clarification

#### 2.8.1. Preparation of Puree

Ripened varieties of three berries viz. strawberries (*Fragaria ananassa*), blueberries (*Cyanococcus*), and raspberries (*Rubus idaeus*) were purchased from a local market in Dublin city. They were thoroughly washed and inspected for any microbial growth and stored at 4 °C for not more than 2 days. The berries were processed using a blender until a smooth puree was obtained. The puree was then filtered using a cheese cloth to separate the pulp from the juice.

#### 2.8.2. Enzyme Treatment

The effectiveness of the purified xylanase for juice clarification was inspected by determining the enzyme dosage and incubation time and temperature and. The dosage used for juice clarification was optimised between the range of 5 to 25 IU/g of fruit pulp. The incubation time and temperature required for optimal reducing sugar release, juice yield, and clarification were determined by conducting experimental trials over ranges of 30 °C to 60 °C for different time periods (3–120 min). Pulp devoid of any enzyme at a retention time of 0 was taken as a control for enzyme dosage and incubation trials, while pulp maintained at 27 °C was used as control for temperature optimisation experiments. After each experiment, the enzymes were deactivated by subjecting the pulp to 80 °C for two minutes, after which the pulp was strained through a cheese cloth to obtain juice after enzymatic treatment.

#### 2.8.3. Determination of Reducing Sugar and Clarity

The clarity in the pulp was measured by determining the percentage transmittance using a UV-VIS spectrophotometer (Perkin Elmer Lambda 900, Bridgeport, Shelton, CT, USA) at a wavelength of 660 nm. Distilled water was used as a reference. Juice clarity was estimated as a measure of percentage transmittance. The release of reducing sugar from puree following enzymatic treatment was measured by using the dinitrosalicylic acid method [15]. For yield measurement, the enzymatically treated fruit pulp was filtered using filter paper (Whatman No. 1, Sigma-Aldrich, Wicklow, Ireland). The filtrate was measured, and the yield was expressed as % *w*/*v* [15].

## 3. Results and Discussion

### 3.1. Optimisation of Fermentation Parameters

SCW was initially subjected to lipid extraction process followed by organosolv pretreatment as a part of the upstream processing strategy. According to our study, 13.4% of the total weight of dry SCW was represented by lipids. Furthermore, SCW was found to be high in hemicellulose content. This fraction of the biomass was enhanced by subjecting it to organosolv pretreatment to enhance xylanase production. Pretreated SCW contained 7.04 g cellulose per 100 g. The hemicellulose sugars in pretreated SCW comprised of 30 g galactose, 3 g arabinose, and 32 g mannose per 100 g pretreated SCW. Minimal amounts of xylose were detected as well. The detailed observations of these experiments have been published in literature elsewhere as part of different studies performed in our facility [13,18].

Solid state fermentation for xylanase production using pretreated SSF was conducted by the application of a Box–Behnken design. Three parameters *viz.* temperature, moisture content, and inoculum size were taken into consideration for optimisation. Table 3 represents the variables, and each variation level. SSF was conducted for 96 h. 

After each trial, the fermented samples were subjected to enzyme extraction process and assayed for xylanase activity and soluble protein content. The results were analysed using Statgraphics Centurion XV software version 15.1.02. The theoretical and observed xylanase activity with respect to the Box–Behnken design has been provided in Table 4. The models were compared based on the coefficient of determination (*R*^2^) and adjusted coefficient of determination (*R*^2^-adj). The *R*^2^ is the regression of sum of squares proportion to the total sum of squares and illustrates the adequacy of a model. *R*^2^ ranges from 0 to 1 and a value closer to 1 indicates that the model is considerably accurate. A value of 99.65% *R*^2^ was observed in the present study while *R*^2^-adj was 97.04%, which illustrates the model adequately fits the data. The results obtained from the Box–Behnken design were fitted to second-order polynomial equation. The polynomial equation for the model is given below:

Xylanase activity (U/g) = − 1.04829 × 10^6^ + 2508.5 × X_1_ + 27204.3 × X_2_ + 17259.2 × X_3_ − 20.65 × X_1_^2^ − 18.2 × X_1_ × X_2_ − 12.15 × X_1_ × X_3_ − 184.813 × X_2_^2^ − 88.125 × X_2_ × X_3_ − 778.0 × X_3_^2^, (3)

where X_1_, X_2,_ and X_3_ represents temperature (°C), moisture content (% (*w*/*w*)), and inoculum size (log no. of spores/g of dry substrate). 

Significant parameters were determined by conducing analysis of variance (ANOVA). ANOVA classifies, and cross classifies statistical results while testing whether the means of the classifications differ significantly. Meanwhile, Fischer’s statistical test of ANOVA determines the influence of each factor and their significance. The F-value is calculated as the ratio of mean square due regression to mean square due to error. The ANOVA table indicated that eight effects have *P*-values less than 0.05, implying that they are significantly different from zero at the 95.0% confidence level and establishing the considerable effect of these coefficients on xylanase activity. Each parameter considered in this study were found to be significant. Latifian, et al. [19] reported temperature effects on enzyme production kinetics, thereby pointing to the importance of temperature control during the fermentation process. Gervais, et al. [20] also reported that water mass transfer is strongly related to the physicochemical control parameters, such as aeration and temperature during a solid-state fermentation process.

**Figure 1:**
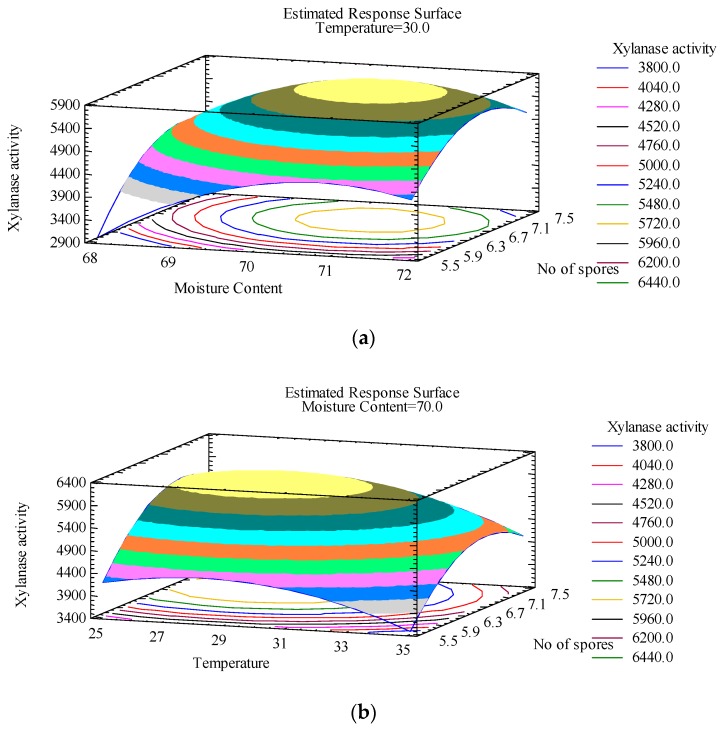
Response surface plots representing the effect of independent variables on xylanase activity: (**a**) The effect of inoculum size (log number of spores) and moisture content (%) when response surface is fixed at temperature = 30 °C; (**b**) the effect of inoculum size (log number of spores) and temperature (°C) when response surface is fixed at moisture content = 70%.

Three-dimensional plots were constructed to understand the interactions between different variables and to determine the optimal level of each variable for maximum response. They provide insight into the interaction between different variables. Response surface plots are generated as a combination of two parameters while keeping the third parameters constant. It has also been reported that water mass transfer is strongly related to the physicochemical control parameters, such as aeration and temperature during a solid-state fermentation process. The 3-D plots related to this optimisation study are provided in Figure 1. 

The plots revealed that there is significant interaction between the different variables considered in the study. The highest point on each plot reveals the optimum value for each variable that facilitates a higher xylanase activity. The model predicted that the best conditions for xylanase production would be maintaining a temperature of 30 °C, an initial moisture content of 70% (*w*/*w*), and an inoculum size of 6.5 × 10^6^ (log number of spores). On performing validation experiment, a high xylanase activity of 5780 ± 0.2 IU/g of dry SCW was recorded. The model predicted a xylanase activity of 6068 IU/g of SCW which suggested minimum disparity (<5%) between predicted and observed values. This also indicated that the model was adequate to predict the optimum values for variables considered for xylanase production adopting SSF mode. Similar observations were reported in other studies investigating lignocellulosic residues for xylanase production via SSF. For example, Pal and Khanum [12] were able to achieve a 4.5-fold increase in xylanase activity using various lignocellulosic substrates and moistening the media to 70%. 

### 3.2. Optimisation of Media Composition

#### 3.2.1. Screening of Media Components

Screening of media components was conducted by employing a Plackett–Burman design of experiments to determine their influence on the yield of xylanase produced by *A. niger* ATCC^®^6275 via solid state fermentation mode. These included organic and inorganic nitrogen sources together with metallic salts amounting to a total of 10 constituents. Table 5 shows the different components and their levels used in Plackett–Burman design. Codes A to J were assigned to each component. 

From the analysis it was determined that SCW supplemented with medium in accordance to trial number 7 resulted in the highest xylanase activity. Yeast extract, MgSO_4_, and KH_2_PO_4_ were present in high titres in this experimental trial. Analysis of variance of the design revealed that these three media components has a significant effect on the production of xylanase by *A. niger.* A regression equation was generated by design software, which related all the media components to the xylanase activity by a first-order polynomial, which is given below:

Xylanase activity (IU/mg) = 4507 − 239 A − 941 B + 275 C − 166 D − 201 E + 308 F + 256 G + 260 I + 125 J.(4)

A model F-value of 15.07 suggested that the model was significant. A determination coefficient (*R*^2^) of 97.66% indicated that the fitted model could be used to predict the media supplements that influenced xylanase production. The analysis of variance showed that yeast extract, MgSO_4_, and KH_2_PO_4_ had pronounced influence on the production of xylanase enzyme (*p* < 0.05). The findings of the Plackett–Burman study were in agreement with other studies based on xylanase production. For example, MgSO_4_ has been reported by several researchers to have a positive influence on xylanase production by bacterial and fungal species alike [21,22]. Furthermore, several studies have reported the importance of KH_2_PO_4_ in xylanase production media. A recent study published by Silva et al. [23] revealed that higher levels of xylanase production was achieved using KH_2_PO_4_ along with corn stover when *Pencillium crostosum* was used as the xylanase producer. Therefore, the other components were neglected, and further studies were conducted employing the three media components to determine the optimum combinations by means of a Box–Behnken design.

#### 3.2.2. Optimisation of Media Composition

A three-parameter, two-level Box–Behken design was adopted for the optimisation of media composition for xylanase production using solid state fermentation mode (Table 6). From the screening study, yeast extract, MgSO_4_, and KH_2_PO_4_ were chosen as the three media supplements for further experiments. 

The parameters and levels were input into statistical software Statgraphics Centurion XV software version 15.1.02., which generated a set of 15 experimental trials. Fermentation was conducted for 96 h in shake flasks containing five grams of pretreated SCW supplemented with media additives. The trials were performed in duplicates and the enzymes extracted from each trial was subjected to xylanase and protein assay protocols. The results obtained have been provided in Table 7. 

The models were compared based on the coefficient of determination (*R*^2^) and the adjusted coefficient of determination (adj-*R^2^*). An *R*^2^ value of 99.07 and an adj-*R*^2^ of 96.26% was observed, which illustrate that the model adequately fitted the data. A second-order polynomial equation was generated by the model into which the data fit, which is given below:

Xylanase activity (IU/g) = 6175.58 + 14326.7 × Y_1_ − 4426.84 × Y_2_ − 18034.1 × Y_3_ − 160357 × Y_1_^2^ + 25463.2 × Y_1_ × Y_2_ − 3500.13 × Y_1_ × Y_3_ + 323.685 × Y_2_^2^ + 29240 × Y_2_ × Y_3_ − 54692.1 × Y_3_^2^, (5)

where Y_1_, Y_2,_ and Y_3_ represents the media components KH_2_PO_4_, yeast extract, and MgSO_4_, respectively. Analysis of variance was conducted to determine the significance of coefficients of the parameters considered in the experimental design. The ANOVA table (Table 8) showed that seven variables were found to be significant considering the *p*-values being less than 0.05 indicating that they are significantly different from zero at the 95.0% confidence level. 

**Figure 2:**
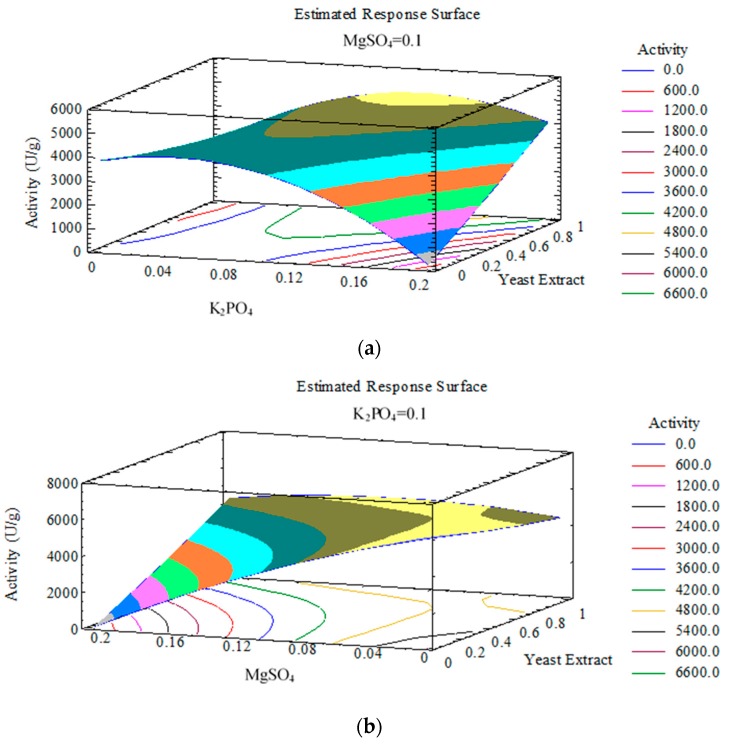
Response surface plots representing the effect of independent variables on xylanase activity: (**a**) The effect of KH_2_PO_4_ and yeast extract on xylanase activity when response surface is fixed at MgSO_4_ = 0.1 g/g of spent coffee waste (SCW); (**b**) the effect of MgSO_4_ and yeast extract on xylanase activity when response surface is fixed at KH_2_PO_4_ = 0.1 g/g of SCW.

This also meant that these values have considerable influence on xylanase activity. Interestingly, the interaction effects (*p* < 0.05) of yeast extract with KH_2_PO_4_ and MgSO_4_ had a positive influence on enzyme activity. Among quadratic coefficients, only yeast extract had a positive effect on maximising the xylanase activity. Three dimensional plots were generated by the model to understand the interactions between different variables and to determine the optimal level of each parameter for maximum response (Figure 2). A maximum xylanase activity of 6495.6 IU/g of activity was achieved by performing solid state fermentation while maintaining the media composition of yeast extract (0.2 g/g of substrate), KH_2_PO_4_ (0.04 g/g of substrate), and MgSO_4_ (0.03 g/g of substrate). The model predicted a maximum xylanase activity of 6225 IU/g of substrate. The experimentally obtained values and the value predicted by the model had little disparity between them (<5%), which suggested that the model was adequate to predict the optimum measures for maximum xylanase activity. As a consequence of the optimisation protocol, a 1.39-fold increase in xylanase activity was observed compared to original media (4649 IU/g of dry SCW).

### 3.3. Purification of Xylanase from *A. niger*

Xylanases are usually purified by a multi-step process that involve non-specific techniques. A higher number of steps adopted in the purification process results in lower yields of the enzyme in most cases [24]. Purification of xylanase needs to be achieved in order to perform complete biochemical analysis and molecular studies. Ion exchange chromatography is a very common technique used to purify xylanase. Several studies have employed DEAE cellulose and DEAE sepharose for the purification of xylanase from fungal species [25,26]. In this study, a three-step purification strategy was adopted to purify *A. niger* ATCC^®^6275 xylanase. A summary of the purification process is detailed in Table 9. 

**Figure 3:**
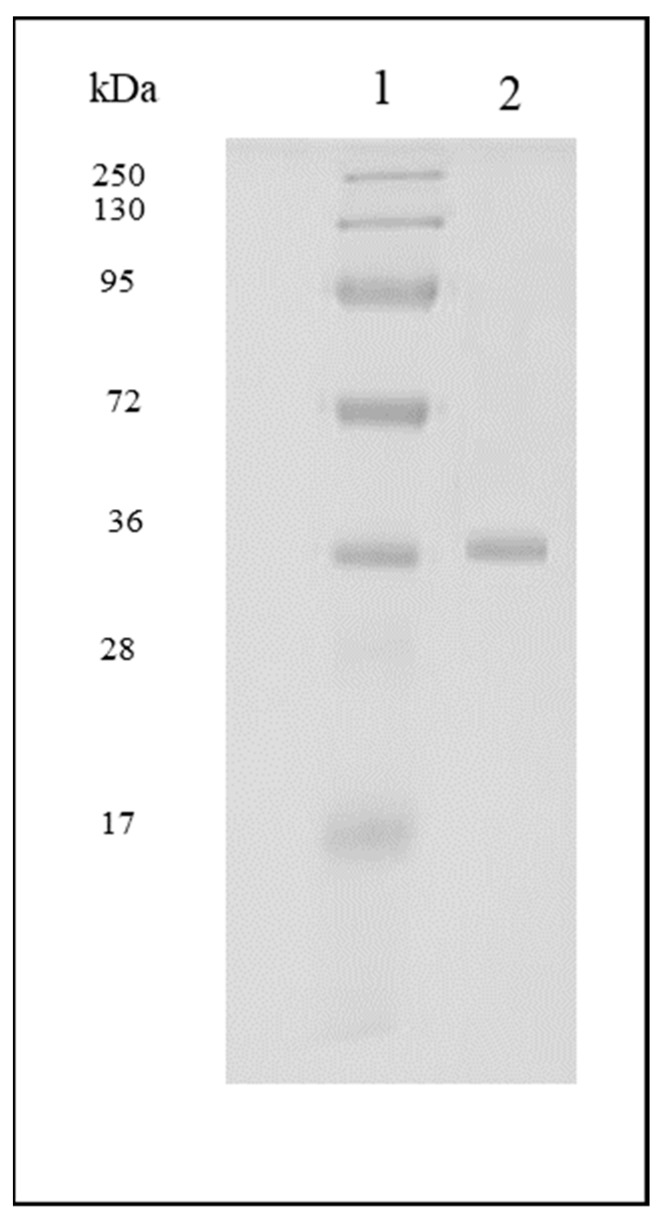
Sodium Dodecyl Sulphate-Polyacrylamide Gel Electrophoresis (SDS-PAGE) (1) molecular size markers; (2) purified xylanase.

The crude enzyme extract exhibited a total activity of 167,250 IU/mL and specific activity of 5009 IU/mg. The crude extract was subjected to (NH_4_)_2_SO_4_ precipitation (65% *w*/*v*) at 4 °C, which resulted in 72% recovery and 3.3-fold purification. The precipitates were then recovered by dissolving them in 0.05 M citrate buffer (pH = 6.0) and subjected to dialysis against the same buffer (1:10 dilution). The buffer was changed several times over a period of 24 h. Subsequently, the partially purified xylanase was concentrated via diafiltration using 10 kDa centrifugal filter unit. This was followed by ion exchange chromatography (DEAE-sepharose fast flow column) involving step elution of 0.5 M KCl after the removal of unbound proteins. The xylanase eluted out at a concentration of 0.2 M. The final enzyme recovery and purification fold of the enzyme observed was 23% and 17.1%, respectively. These results were consistent with other studies that employed purification strategies. Pal and Khanum [27] conducted an extensive study to purify and characterise thermostable xylanase from *A. niger* DFR-5. A four-step strategy was employed, which encompassed ion-exchange chromatography followed by gel permeation chromatography. A final yield of 38.9% and purification fold of 36.97% was reported in this study. In another study involving *A. niger* xylanase cloned into *E. coli*, a three-step purification measure was adopted involving a Ni-NTA resin and FPLC Mono-Q chromatography to obtain a high yield of 67.5% [28]. The molecular mass of purified xylanase was determined by relative mobility of standard proteins on SDS-PAGE. The xylanase enzyme appeared as a single protein band in the gel suggesting its homogenous monomeric nature with a single polypeptide chain (Figure 3). The relative molecular mass of denatured xylanase was estimated using 10% SDS-PAGE (Sodium Dodecyl Sulphate-Polyacrylamide Gel Electrophoresis) and appeared to be ~36 kDa. The molecular weight of xylanase derived from *A. niger* has been reported to vary from 19 kDa to 86 kDa [29]. 

### 3.4. Effect of pH and Temperature on Enzyme Activity

The optimum pH for activity and stability of an enzyme are important parameters in determining its potential commercial applications. Xylanases derived from *A. niger* have been reported to be functional at an acidic-to-neutral pH range [30]. The enzyme was moderately stable at a broad pH range extending from pH 2.0–8.0. The optimum pH of xylanase was determined to be 5.0. High activity at this pH is a clear indication that the xylanase investigated in this study may find application in juice clarification [14]. Extensive pH stability studies were conducted to analyse the robustness of the xylanase. The enzyme was preincubated in a pH range of 4.0–6.5 and the activity was measured at pH 5.0. No decline in activity was observed by this process. However, incubating the enzyme at pH < 4.0 or pH > 6.5 resulted in decreased activity. This might be due to the irreversible inactivation of the enzyme. Factors such as pH and temperature have been known to bring permanent spatial conformational changes in enzymes [31]. The stability of the xylanase was assessed at various temperatures ranging from 20 °C to 50 °C. The optimum temperature for maximum enzyme activity was found to be 35 °C. The enzyme showed no signs of thermostability as increasing the temperature beyond 40 °C resulted in a decrease in xylanase activity. Xylanases derived from *A. niger* ATCC 6275 have been reported to exhibit high activity only in the mesophilic range [32].

### 3.5. Application of Xylanase in Fruit Juice Enrichment

The potential application of purified *A. niger* xylanase was investigated for the enrichment of strawberry, blueberry, and raspberry pulps. Enzyme dosage, incubation time, and incubation temperature were the three variables included in this study. The efficacy of the enzyme as an enhancement measure was examined by estimating the variations in juice yield (%), clarity (%), and reducing sugars (%). 

#### 3.5.1. Effect of Enzyme Dosage

The optimum enzyme dosage for clarification of the different fruit pulp juices was determined by varying the concentration of enzymes (Figure S1). Accordingly, a range of concentrations spanning from 5 to 25 IU/gram fruit pulp (gfp) was taken in consideration in the study. The fruit pulp was incubated with different concentrations of the enzymes at room temperature (25 °C) for 60 min. Fruit pulp treated with heat inactivated xylanase was taken as 100% for each experiment. The optimum enzyme dosage with respect to reducing sugar content, clarity, and juice yield differed with respect to each variety of fruit pulp. For example, the reducing sugar content, clarity, and yield increased as the dosage was increased until 10~15 IU/gfp. While a dosage of 10 IU/gfp was found to be ideal for strawberry, 15 IU/gfp was found to be the optimum for blueberry and raspberry with respect to achieving higher reducing sugar content, yield, and clarity. Fruits commonly consist of a primary cell wall, which is composed of cellulose, hemicellulose, embedded lignin, and in certain cases, pectin. The middle lamella functions as the connecting agent that holds the cells together [33]. Enzymatic treatment of fruit pulp results in the hydrolysis of the cell wall making juice recovery easier. Increasing the enzyme dosage results in clarification of fruit juice due to the exposure of charges protein particles within. These protein particles clump together due to electrostatic forces and settle down freely [34]. Progressive increase of enzyme dosage ensures that all the hemicellulose is eventually digested, leaving the proteins in the pulp leading to decrease in clarity. This might be the reason why high enzyme dosage is detrimental to the clarification of fruit pulp [35]. 

#### 3.5.2. Effect of Temperature

Temperature is an important variable when considering the application of enzymes for commercial purposes. The incubation temperature of enzymactic enrichment of fruit pulp was studied (Figure S2). A fixed dosage of 10 IU/gfp was considered for this study while varying the temperature between 30–60 °C. The enzymatic treatment was performed for 60 min. A control experiment maintaining the same dosage and incubation time was conducted at room temperature (25 °C). A temperature of 35 °C was found to the best with respect for all the three fruit pulp varieties (strawberry, blueberry, and raspberry) tested in terms of reducing sugar content, clarity, and yield. This was in agreement with the optimum temperature of the xylanase determined earlier in this study. Enzyme reaction rates increase as temperature increases and, as a result, the rate of clarification increases until the enzyme is denatured by heat. The yield and clarification of the fruit pulp increases as temperature increases due to the digestion of polysaccharides by xylanase. This decreases the water holding capacity of the fruit pulp subsequently increasing the yield and clarity of the fruit pulp [35].

#### 3.5.3. Effect of Incubation Time

Optimisation of incubation time for enzymatic treatment of fruit pulp was investigated (Figure S3). A range of time periods spanning from 30 min to 120 min was considered for this study. Dosage was fixed at 10 IU/gfp and experiments were done at room temperature (25 °C). An experimental trial with fruit pulp treated by xylanase for 0 min was taken as control (100% benchmark). Increase in incubation time resulted in an initial increase in clarity of the fruit pulp. However, extended incubation time was not recommended as it led to the decrease in clarity. This may be due to the formation of haze particles by protein–carbohydrate of protein–tannin complexes. The optimum incubation time for strawberry fruit pulp was found to be 90 min while, a time between 60 min and 90 min was preferable for blueberry pulp. An optimum incubation time of only 60 min was required for raspberry fruit pulp.

## 4. Conclusions

In the present work, we have demonstrated how optimisation of culture conditions for *A. niger* and media by Box–Behnken design can lead to substantial increase in xylanase production. From our observations, it was evident that SCW can be an excellent carbon source for the production of fungal xylanase. Statistical analysis showed that all three parameters involved in fermentation have a significant effect on xylanase production. Under optimised conditions, a maximum activity of 6495.6 IU/g of dry SCW was achieved. The xylanase produced by *A. niger* ATCC^®^ was found to be an excellent fruit juice clarification agent. Xylanase yield can be improved by involving a mutant strain. Process scale-up and further recovery techniques need to be investigated to attain commercial feasibility.

## Figures and Tables

**Table 1:** Variables and level for Box–Behnken design for the optimisation of physical parameters.

Factors	Coded Symbols	Basic Level	Variation Level	Value of Factor	Coded Value
Temperature	X_1_	30	5	25	−1
				30	0
				35	+1
Moisture content (%)	X_2_	70	2	68	−1
				70	0
				72	+1
Log (number of spores)	X_3_	6.5	1	5.5	−1
				6.5	0
				7.5	+1

**Table 2:** Nutrient supplements for the screening of nutrients using Plackett–Burman method.

Nutrient Code	Compound	(+) Level (%) (g/g of Dry Substrate)	(−) Level (%) (g/g of Dry Substrate)
A	KH_2_PO_4_	0.2	0.1
B	NaCl	0.1	0.05
C	MgSO_4_	0.1	0.05
D	Yeast Extract	0.2	0.1
E	Peptone	0.2	0.1
F	(NH_4_)_2_SO_4_	0.2	0.1
G	NH_4_Cl	0.2	0.1
H	CaCl_2_	0.1	0.05
I	FeCl_3_	0.1	0.05
J	KCl	0.1	0.05

**Table 3:** Variables and level for Box–Behnken design for the optimisation of physical parameters.

Independent Variables	Coded Symbols	Levels		
		−1	0	+1
Temperature	X_1_	25	30	35
Moisture content (%)	X_2_	68	70	72
Log (number of spores)	X_3_	5.5	6.5	7.5

**Table 4:** Box–Behnken experimental design for SSF optimisation employing three independent variables, experimental, and predicted values for xylanase activity.

Trial No.	Temperature (°C)	Moisture Content (%)	Log (No. of spores)	Observed Xylanase Activity (U/g)	Predicted Xylanase Activity (U/g)
1	25	68	6.5	4215	4281
2	30	68	7.5	4653	4572
3	30	72	5.5	4113	4194
4	25	70	7.5	5504	5520
5	35	72	6.5	4358	4293
6	35	70	5.5	3477	3461
7	25	70	5.5	4190	4174
8	25	72	6.5	5557	5491
9	30	70	6.5	5724	5724
10	30	70	6.5	5724	5724
11	35	70	7.5	4548	4564
12	30	72	7.5	5016	5066
13	35	68	6.5	3744	3810
14	30	68	5.5	3045	2995
15	30	70	6.5	5724	5724

**Table 5:** Experimental design for the screening of nutrients using Plackett–Burman method.

Blocks	A	B	C	D	E	F	G	H	J	Activity/g of Dry Substrate (U/g)
1	1	−1	1	1	−1	1	−1	−1	−1	5451
2	−1	−1	−1	1	1	1	−1	1	1	5692
3	−1	1	1	1	−1	1	1	−1	1	4281
4	1	1	−1	1	−1	−1	−1	1	1	1697
5	−1	−1	1	1	1	−1	1	1	−1	4467
6	1	1	1	−1	1	1	−1	1	−1	3488
7	1	−1	1	1	−1	−1	1	1	1	6034
8	−1	1	1	−1	1	−1	−1	−1	1	3611
9	−1	1	−1	−1	−1	1	1	1	−1	4860
10	1	1	−1	1	1	−1	1	−1	−1	3457
11	1	−1	−1	−1	1	1	1	−1	1	4118
12	−1	−1	−1	−1	−1	−1	−1	−1	−1	4567

**Table 6:** Variables and level for Box–Behnken design for the optimisation of nutrient supplements.

Independent Variables	Coded Symbols	Levels		
		−1	0	+1
Yeast Extract	Y_1_	0	0.5	1.0
K_2_HPO_4_	Y_2_	0	0.1	0.2
KCl	Y_3_	0	0.1	0.2

**Table 7:** Box–Behnken experimental design for SSF optimisation employing three independent variables, experimental, and predicted values for xylanase activity.

Trial No.	K_2_HPO_4_ (g/g of SCW)	Yeast Extract (g/g of SCW)	MgSO_4_ (g/g of SCW)	Xylanase Observed (U/g)	Xylanase Theoretical (U/g)
1	0.2	0.0	0.1	56	206
2	0.2	1.0	0.1	4149	4120
3	0.1	0.5	0.1	4222	4222
4	0.1	0.0	0.0	6065	6005
5	0.0	0.5	0.2	1083	1173
6	0.2	0.5	0.0	3130	3040
7	0.1	0.0	0.2	259	140
8	0.1	1.0	0.2	4371	4431
9	0.0	0.5	0.0	4012	4043
10	0.0	1.0	0.1	2796	2646
11	0.1	0.5	0.1	4222	4222
12	0.1	1.0	0.0	4329	4448
13	0.0	0.0	0.1	3796	3825
14	0.1	0.5	0.1	4222	4222
15	0.2	0.5	0.2	61	30

**Table 8:** Box–Behnken experimental design for SSF optimisation employing three independent variables, experimental, and predicted values for xylanase activity.

Source	Sum of Squares	Degrees of Freedom	Mean Square	F-Ratio	*p*-Value
Y_1_	2.30 × 10^6^	1	2.30 × 10^6^	114.26	0.0001
Y_2_	3.74× 10^6^	1	3.74 × 10^6^	185.62	0.0002
Y_3_	1.73 × 10^7^	1	1.73 × 10^6^	858.69	0.0003
Y_1_ Y_1_	9.49 × 10^6^	1	9.49 × 10^6^	471.45	0.0324
Y_1_ Y_2_	6.48 × 10^6^	1	6.48 × 10^6^	321.95	0.0021
Y_1_ Y_3_	4900.35	1	4900.35	0.24	0.6427
Y_2_ Y_2_	24178.1	1	24178.1	1.2	0.3232
Y_2_ Y_3_	8.55 × 10^6^	1	8.55 × 10^6^	424.54	0.0041
Y_3_ Y_3_	1.10 × 10^6^	1	1.10 × 10^6^	54.84	0.0007
Total error	100695	5	20139		
Total (corr.)	4.88 × 10^6^	14			

**Table 9:** Purification of xylanase from *A. niger.*

Step	Total Activity (IU)	Total Protein (mg)	Specific Activity (IU/mg)	Purification Fold	Yield
Crude extract	167,250	5009	33	1.0	100
Ammonium sulphate	155,309	1412	110	3.3	72
Ultrafiltration	93,336	173	540	16.2	56
Ion-exchange chromatography	39,036	68	572	17.1	23

## Supplementary Materials

The following are available online at https://www.mdpi.com/2304-8158/8/11/585/s1, Figure S1: Effect of enzyme dosage on juice enrichment with respect to reducing sugar content, clarity and yield; Figure S2: Effect of temperature on juice enrichment with respect to reducing sugar content, clarity and yield; Figure S3: Effect of incubation time on juice enrichment with respect to reducing sugar content, clarity and yield.
