# Supplementary material for: Spent Coffee Waste as a Potential Media Component for Xylanase Production and Potential Application in Juice Enrichment
Source: Foods. 2019 Nov 17;8(11):585. doi: 10.3390/foods8110585 (PMC6915662; doi:10.3390/foods8110585)
Supplement: Supplementary file 1 [file foods-08-00585-s001.pdf]

Supplementary Data

Article

Spent Coffee Waste as a Potential Media Component for Xylanase Production and Potential Application in Juice Enrichment

Rajeev Ravindran [1,2], Gwilym A. Williams [2] and Amit K. Jaiswal [1,*]

[1] School of Food Science and Environmental Health, College of Sciences and Health, Dublin Institute of Technology, Cathal Brugha Street, Dublin D01 HV58, Ireland; rajeev.ravindran@mydit.ie

[2] School of Biological Sciences, College of Sciences and Health, Dublin Institute of Technology, Kevin Street, D08 NF82 Dublin, Ireland; gwilym.williams@TUDublin.ie

* Correspondence: email: amit.jaiswal@TUDublin.ie; akjaiswal@outlook.com; Tel: +353-1402-4547

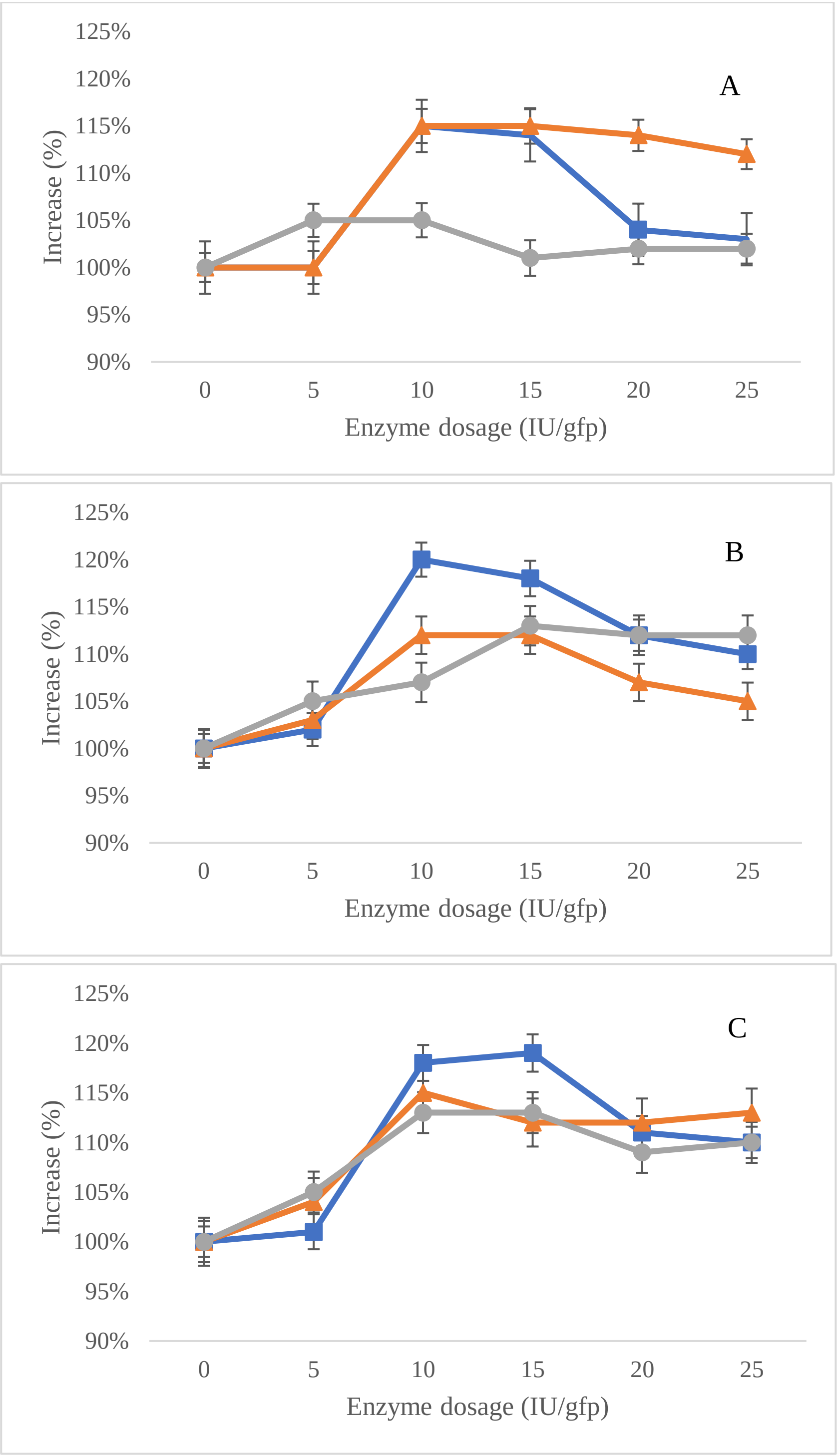

Figure S1. Effect of enzyme dosage on juice enrichment with respect to reducing sugar content (■) clarity (▲) and yield (●) (A) of strawberry, (B) blueberry and (C) raspberry

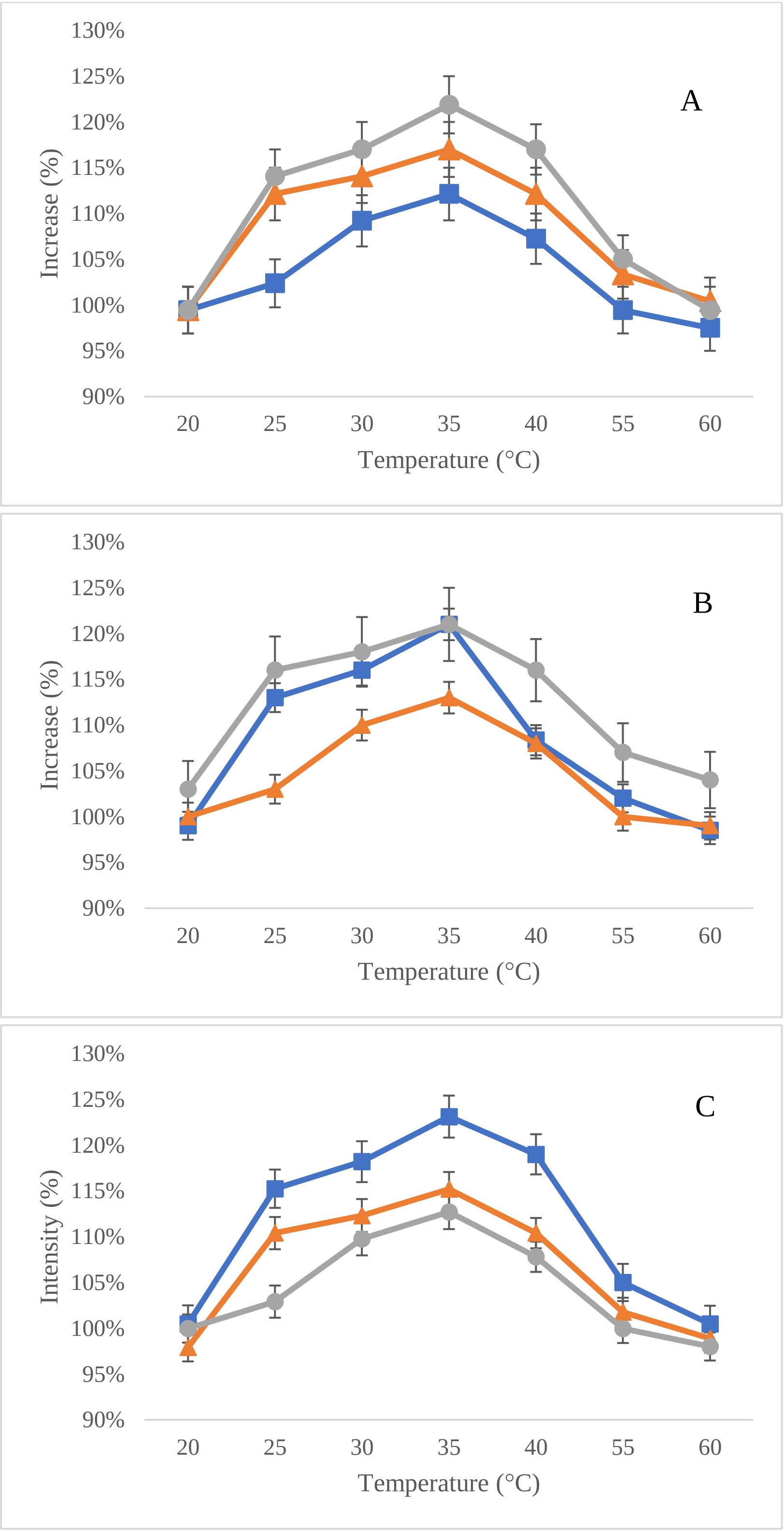

Figure S2. Effect of temperature on juice enrichment with respect to reducing sugar content (■) clarity (▲) and yield (●) of (a) strawberry (b) blueberry and (c) raspberry

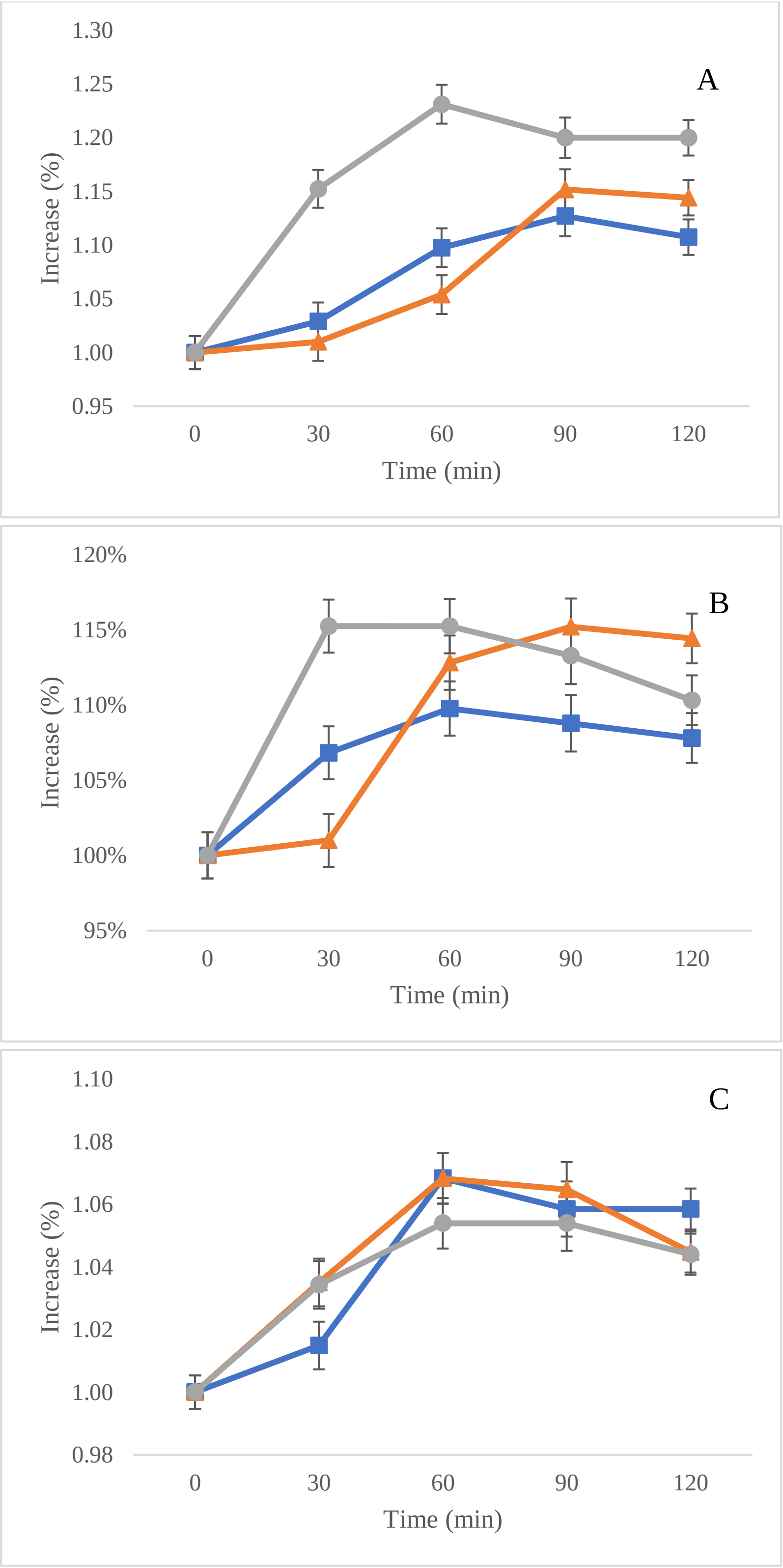

Figure S3. Effect of incubation time on juice enrichment with respect to reducing sugar content (■) clarity (▲) and yield (●) of (a) strawberry, (b) blueberry and (c) raspberry
